# Patient pathways to PrEP persistence: a qualitative resilience-focused journey mapping study with cisgender Black and Latino men who have sex with men and clinical PrEP staff at a federally qualified health center

**DOI:** 10.3389/frhs.2026.1794839

**Published:** 2026-07-10

**Authors:** Steven Meanley, Emily A. Arnold, Louis Listerud, Tyler Burgese, Alana Richards, Blake Kosciow, Emily Hiserodt, Amelia Parchment, Danielle Rocha, José A. Bauermeister, Alida M. Bouris

**Affiliations:** 1 Department of Family and Community Health, University of Pennsylvania School of Nursing, Philadelphia, PA, United States; 2 Eidos LGBTQ+ Health Initiative, University of Pennsylvania School of Nursing, Philadelphia, PA, United States; 3 Center for AIDS Prevention Studies, Department of Medicine, University of California San Francisco School of Medicine, San Francisco, CA, United States; 4 Philadelphia FIGHT, Philadelphia, PA, United States; 5 Crown Family School of Social Work, Policy, and Practice, The University of Chicago, Chicago, IL, United States

**Keywords:** care navigation, gay and bisexual men, patient journey, PrEP, racial and ethnic-minoritized, resilience

## Abstract

**Background:**

Persistence in pre-exposure prophylaxis (PrEP) use remains inequitably implemented among cisgender Black and Latino men who have sex with men (BLMSM) who could benefit from PrEP. Despite multilevel social barriers across the PrEP care continuum, many BLMSM mitigate or overcome these barriers to sustain their PrEP use, suggesting underlying resiliencies that can strengthen PrEP programs and policies.

**Methods:**

We conducted qualitative journey mapping interviews with 16 PrEP-sustained BLMSM and 7 clinical PrEP staff (medical and social/outreach services) at a federally-qualified health center (FQHC) in Philadelphia, Pennsylvania (United States). Interviews explored BLMSM's PrEP trajectories, elucidating which processes and resources supported BLMSM's ability to overcome barriers (e.g., job transitions, health insurance coverage).

**Results:**

PrEP trajectories included cyclical knowledge-building and contemplation, immediate linkage upon learning about PrEP when accessing health services, brand and modality transitions, and periods of disengagement followed by re-linkage. Structural barriers related to cost, insurance coverage, pharmacy access, clinic hours, housing and employment instability, and medical burnout and fatigue were identified. BLMSM and PrEP staff identified key facilitators supporting barrier reduction, such as affirming provider communication, co-located phlebotomy, transportation assistance, and problem-solving adherence challenges. Participants emphasized PrEP coordinators as being central to support PrEP service navigation, outreach for PrEP discontinuers, problem-solving financial challenges, and liaising between insurance companies and pharmacies.

**Discussion:**

Our findings illuminate how resilience processes (i.e., overcoming barriers) and patient-centered, team-based support within an FQHC can transform common disruptions into opportunities for sustained or renewed PrEP engagement, advancing resilience-informed PrEP engagement models that advocate for practice-oriented solutions and future implementation research.

## Introduction

Pre-exposure prophylaxis (PrEP) refers to the uptake of antiretroviral medications by people without HIV to greatly reduce their risk of acquiring of HIV through sexual contact or injection drug use. PrEP is currently administered via oral formulations and long-acting injectables (LAI) (1). As a biobehavioral HIV prevention strategy, PrEP has been critical toward efforts to end the HIV epidemic in disproportionately-affected populations (2). When taken as recommended, PrEP can reduce the risk of HIV transmission by up to 99%, highlighting need to prioritize support for its access, uptake, and sustained use (3). The current gap between clinical efficacy and real-world implementation highlights a central challenge to maximizing PrEP's impact on HIV prevention. As of 2022, only 36% of people in the United States who could benefit from PrEP had been prescribed the medication, including only 13% and 24% among Black and Hispanic populations, respectively (4). Despite being highly effective, PrEP's impact depends on access, engagement, and sustained use (5). Since PrEP's early rollout, substantial efforts have been made to support PrEP use among men who have sex with men (MSM) given this community's historical HIV burden. Current estimates indicate that the lifetime risk of an HIV diagnosis among all MSM is 1 in 7; however, this statistic masks the 1 in 3 lifetime risk among Black MSM and 1 in 5 among Latino MSM, while the risk decreases to 1 in 15 among non-Hispanic White MSM (6, 7).

Across clinical and community settings, PrEP remains underutilized among BLMSM who could benefit from uptake the most (e.g., those who inconsistently use condoms) (8, 9). PrEP use continues to increase among all MSM, yet uptake in Black MSM (38%) and Latino MSM (43%) continues to lag behind non-Hispanic White MSM (51%) (10). Similarly, prior observational studies have exhibited mixed findings on racial and ethnic disparities in rates of PrEP care retention, adherence (i.e., taking PrEP as prescribed), and persistence (i.e., sustained, long-term PrEP use) (11–13). Large, national cohort studies depict similar PrEP discontinuation rates across racial and ethnic backgrounds but have found noticeable differences in taking PrEP as prescribed, particularly in the context of daily-oral PrEP (12, 14). Post-uptake challenges reflect multilevel barriers across various settings such as insurance instability, economic constraints, lack of affirming providers, stigma, and medical mistrust shaped by prior discriminatory experiences (15–22). Disengaging with PrEP care, particularly when PrEP-indicated (i.e., having a sero-negative status along with at least one of the following: having a sexual partner with HIV, having multiple partners, recently engaging in condomless anal intercourse, or having a recent bacterial diagnosis of a sexually transmitted infection) increases one's risk for HIV acquisition three to fourfold (23, 24). Equitable PrEP strategies must address structural barriers, strengthen trust in healthcare, and develop interventions with BLMSM that reflect their lived experiences.

Because federally-qualified health centers (FQHC) are structured to deliver ongoing primary care and support services to patients facing these very barriers, they are an ideal setting for examining how BLMSM sustain or resume PrEP use over time and for designing equity-focused interventions (25, 26). FQHCs occupy a distinct niche in this landscape as community-based, primary care safety-net clinics that provide comprehensive services regardless of ability to pay, in contrast to hospital-based systems that are often more episodic, specialty-driven, and tied to insurance status (9). Understanding PrEP navigation among patients receiving care in an FQHC service delivery model offers a window into how PrEP engagement can be equitably supported among individuals from marginalized and underserved backgrounds.

The PrEP care continuum outlines key stages of engagement, including awareness, willingness, initiation, adherence, and retention in care (27). Though widely acknowledged as nonlinear engagement, the PrEP care continuum is often presented as a linear sequence, undermining its real-world complexity (28, 29). The continuum identifies stage transitions but reveals little about patient-centered decision-making processes, contextual influences, or turning points that may help to explain why an individual may advance or stall across stages over time. Qualitative methods are particularly well suited to address this challenge, elucidating how PrEP-indicated individuals experience and navigate PrEP-related decisions, barriers, and support systems across and between stages. Additionally, incorporating provider perspectives on patient-centered practices could further highlight how clinical communication, relational dynamics (e.g., trust building through continuity in care), and structural constraints shape how PrEP options are discussed, interpreted, and acted upon. Prevention experts have called for expanding continua to include alternate trajectories that account for patterns such as temporary interruptions, seasonal fluctuations, or re-initiation following changes in life or care access (30). Furthermore, expanded conceptualizations of the PrEP care continuum may inform analytic scale-up for large-scale insights on PrEP trajectories (e.g., metrics, modeling) as new PrEP formulations and modalities become available (31).

Qualitative journey mapping is a method that leverages patient or participant data (i.e., through in-depth interviews) to visually and narratively depict temporal healthcare engagement by tracing decision points, emotional responses, barriers, and resources over time (32). Widely used in chronic disease management, behavioral health, and patient experience research, applications in PrEP remain underutilized (33–36). This approach can capture how PrEP engagement unfolds in practice, revealing nonlinear patterns, emotional and relational drivers, and key moments when structural barriers are most acute, insights that are not easily observed through traditional models. In a recent qualitative journey mapping study, Lane and colleagues elucidated how a two-level system (system 1: emotional; system 2: cognitive) for PrEP-related decision-making was used among a predominantly non-Hispanic White MSM sample receiving PrEP services at an academic medical healthcare system (37). They highlighted when (e.g., information gathering vs. post-uptake stages) along the PrEP care continuum salient attributes (e.g., feeling protected, taking ownership of one's health, side effects, cost, etc.) emerged. Though this study contributes critical insight into PrEP decision-making processes, less attention was paid to strategies for supporting common perceived PrEP access barriers. This gap warrants the implementation of journey mapping methods within low-barrier clinical paradigms, like FQHCs, highlighting how clinic-based patient-provider interactions, available in-house resources, and community partnerships can be leveraged to support BLMSM access, sustain, and if necessary, re-engage in PrEP, when faced with complex barriers and challenges (38).

Journey mapping can foreground resilience by tracing how people adapt, enlist support, and re-engage with care in response to shifting circumstances (39). Predominantly applied in mental health research, prior studies have operationalized resilience as an individual-level psychological trait as well as the presence of supportive or health-promoting factors when faced with adversity (40–42). Conceptualizations of resilience have continued to evolve in public health research to account for the adaptive processes in which individuals achieve desired (or avoid negative) health outcomes when their personal strengths (e.g., coping skills, motivation) interact with accessible, meaningful social and environmental resources (e.g., supportive relationships and contexts) to overcome multilevel barriers or challenges (40). In this definition, resilience encapsulates processes at the individual-, clinic-, and systems-levels. Furthermore, centering resilience in PrEP engagement requires moving beyond deficit-based narratives and highlighting how individuals achieve and health systems support PrEP persistence, if applicable, while actively avoiding, confronting, and overcoming challenges (40). Journey maps may capture resiliencies that are relevant among BLMSM, including relational and community-based support, cultural resourcefulness, and problem-solving strategies developed within structural constraints (38). As demonstrated in HIV care contexts, resilience is visible not only in patient actions, but also among operations-focused responses within FQHCs, such as flexible scheduling, telehealth outreach, financial assistance navigation, and persistent follow-up with patients who miss visits or experience lapses (43). Yet most PrEP research underrepresents how clinics adapt care to meet patient needs and emphasizes barriers over agency or perceived capacity. By visualizing temporal patterns and decision points in an FQHC context, journey maps can reveal patient and clinical strategies that succeed in practice and inform recommendations for providers and programs seeking to strengthen PrEP persistence in safety-net primary care.

Our study aimed to advance and deepen an understanding of PrEP engagement among BLMSM receiving PrEP services at an FQHC in Philadelphia, Pennsylvania (United States), attending to patient-centered and resilience-informed processes (44). In 2023, 58.2% of new diagnoses were observed among MSM, 64.5% among individuals 20–39 years of age, 61.6% w in Non-Hispanic Black communities, and 17.7% were observed in Hispanic/Latine communities (45). Among new cases in MSM, more than 75% were observed in BLMSM (45). Through qualitative journey mapping with patients, we explored and visualized transitions across the PrEP care continuum, describing how BLMSM navigated salient barriers. In addition, we interviewed clinical FQHC-based PrEP support staff to contextualize patient experiences and ascertain system-level strengths and opportunities for improvement and sustainment.

## Methods

Data for the current study come from the Patient Pathways to PrEP Persistence (P4) Study, a community-academic collaboration between the clinical research infrastructure housed within an FQHC, Philadelphia FIGHT, and a research team at the University of Pennsylvania. The primary objective of the study was to explore cisgender BLMSM's processes for overcoming barriers to PrEP engagement along the PrEP care continuum. The study employed a qualitative descriptive design, an appropriate approach for soliciting diverse, subjective, and appraised experiences navigating healthcare interventions (46). All subsequently described methods and procedures were approved by the Institutional Review Boards at Philadelphia FIGHT and the University of Pennsylvania.

### Participant recruitment, enrollment, and data collection

The P4 Study employed one-time, semi-structured patient journey mapping interviews with cisgender BLMSM. Prospective participants were eligible if they: (1) were a current patient at Philadelphia FIGHT, (2) lived or stayed in a Philadelphia Metropolitan Area ZIP Code, (3) self-identified as a cisgender man, (4) self-identified as Black and/or Hispanic/Latino, (5) were between the ages of 18 and 39 [to align with local HIV incidence data] (45), (6) were currently taking PrEP (daily-oral, episodic, or bimonthly LAI), (7) had been retained in PrEP care for at least two concurrent visits irrespective of PrEP modality, (8) had condomless anal intercourse with at least one male partner in the past year, and (9) were willing to complete a qualitative interview in English or Spanish. We purposively sampled BLMSM to ensure representation of those who reported ever having to discontinue their PrEP use (i.e., intentional or unintentional PrEP discontinuation). Participant recruitment activities were conducted in-house by Philadelphia FIGHT research staff. BLMSM were pre-screened using electronic medical records by FIGHT research staff then contacted during PrEP follow-up appointments, via SMS-texting, phone calls, or patient portal messaging. Once contacted, BLMSM were verbally-screened based on eligibility criteria.

To contextualize patient perspectives, we also conducted one-time, semi-structured interviews with currently-employed clinical PrEP staff at Philadelphia FIGHT. To participate, staff had to report: (1) providing direct, patient-facing services to patients who identify as cisgender BLMSM (e.g., medical providers prescribing PrEP, nursing staff, medical case management staff, social work staff, HIV testers, and PrEP coordinators and (2) being employed in an HIV prevention role for at least 6 months. PrEP staff were contacted via email by FIGHT research staff.

All prospective participants (BLMSM and PrEP team staff) were directed to complete an electronic informed consent form and a screener verification survey programmed on a HIPAA-compliant REDCap survey housed within the University of Pennsylvania. In addition, BLMSM participants signed an authorization form for release of patient information. After providing consent, research staff at the University of Pennsylvania coordinated with participants to schedule qualitative interviews. A total of 26 participants were recruited and completed informed consent procedures from Philadelphia FIGHT, including 19 BLMSM and 7 PrEP staff members. One participant was deemed ineligible given their non-cisgender identity and after four contact attempts, two other BLMSM were unable to be contacted and were considered lost-to-follow-up, leaving our final analytic sample to 16 BLMSM and 7 PrEP staff members. All interviews were conducted one-on-one between April and November 2024 on Zoom for convenience and to minimize travel burden. Study interviewers included two researchers trained in public health, HIV health equity research, and sexual and gender minority health. Both interviewers identify as queer (including one MSM) and BIPOC (Black, Indigenous, and People of Color) and reflected the age range of BLMSM participants. Interviews lasted between 30 and 90 min. BLMSM and PrEP staff were compensated $75 for their time and lived expertise.

### Interview guides

Journey mapping methods enabled the visualization and analysis of temporal care pathways, decision points, and resilience behaviors, focusing on both barriers and facilitators impacting PrEP engagement in real-world settings. For BLMSM participants, the semi-structured interview guide was designed to trace participants' PrEP care trajectories, sectioning questions and probes by stage (Part 1—*Becoming aware, learning, and considering taking* PrEP, Part 2—*Talking to a provider and preparing to get on PrEP*, Part 3—*Starting PrEP*, Part 4—*Maintaining PrEP use*) allowing individuals to narrate their experiences across key milestones. Part 4 solicited questions about any experiences with PrEP disruptions, discontinuation, and re-engagement. All interviews were conducted by SM and LL. The study interviewers used visual guides aligned with the stages described for Parts 1 through 4 presented as PowerPoint slides. Each slide was presented and left visible upon transitions to subsequent sections of the interview. For example, the Part 2 slide was presented to participants once Part 1 discussions were completed and left visible until the interview transitioned to Part 3 questions. The interview leveraged timeline-based questioning to anchor participant responses (e.g., When did you first hear about PrEP? How long after deciding to pursue PrEP did you make an appointment with a provider? What made making an appointment challenging? What made making an appointment easy?). BLMSM were asked to reflect on salient barriers, if relevant, across specific stages (e.g., identifying a PrEP provider) and to describe how they overcame those specific challenges. Similar to the patient interview guide, PrEP staff's interview questions were anchored across each stage of the PrEP care continuum from PrEP awareness to maintenance. PrEP staff were asked about their perceptions of BLMSM's salient barriers and strategies leveraged across the PrEP care continuum. Additionally, PrEP staff were asked to describe their protocol for introducing and supporting PrEP engagement.

### Data analysis

All interviews were audio recorded, transcribed verbatim, and quality-checked by the research team to ensure accuracy and contextual fidelity. Transcripts were uploaded into Dedoose qualitative analytic software and coded using an abductive approach that incorporated both theory-informed constructs and emergent concepts (47, 48). Initial codes were developed deductively from the interview guide domains (e.g., PrEP care continuum stages) and resilience-informed processes (e.g., barriers and processes of overcoming them). The codebook was supplemented by inductive codes emerging from the initial transcripts (e.g., impact of mental health and substance use on PrEP engagement). Four transcripts (two from BLMSM participants, two from PrEP Staff) were initially coded together as a team (SM, TB, AR, LL, BK) to build consistency in interpretation and code application (49). Coding disagreements were reconciled as a team, resulting in a finalized, structured codebook that guided subsequent analysis. The remaining transcripts were each randomly assigned to be coded by two team members. Within these pairs, coding decision discrepancies were resolved through discussion.

Interviews were analyzed using codebook thematic analysis to identify patterns of PrEP engagement across the care continuum, focusing on participants' knowledge mobilization, decision-making processes, key turning points, and resilience strategies (i.e., how BLMSM overcame barriers and how PrEP staff supported these processes) (50). We selected codebook thematic analysis because it provided a systematic and transparent approach for analyzing our qualitative dataset while allowing both deductive attention to the study aims and inductive identification of emergent patterns. In parallel, the first author reviewed coded transcripts to identify common patterns from the codebook thematic analysis and sort them into trajectory types (e.g., linear progression, cyclical disengagement/re-engagement, modality transitions) based on recurrent codes representing movement across PrEP care continuum stages and resilience processes. Cross-participant comparisons of coded excerpts revealed shared thematic patterns (e.g., common turning points, barrier/facilitator codes), which were synthesized into composite journey maps illustrating prototypical pathways observed across participants rather than individual case narratives (32, 38). Journey maps were reviewed, confirmed, and reconciled with the other four study coders.

## Results

### Participant characteristics

Ten (58.8%) BLMSM were under 30 years of age and six (41.2%) were between 30 and 39 years-old (Table 1). Eleven (68.8%) participants self-reported as Black and six (37.5%) as Hispanic/Latino non-exclusively. Thirteen (81.3%) BLMSM self-identified as gay, one (6.2%) as bisexual, one (6.2%) as pansexual, and one (6.2%) as queer. Thirteen (81.3%) BLMSM had at least a two-year college or vocational education. Four participants (25%) had no health insurance, and two (12.5%) participants reported being unstably housed, but all (100%) participants reported that their basic financial needs were being met. In terms of PrEP use (Table 2), 10 (58.8%) BLMSM were on the bimonthly LAI PrEP and six (41.2%) were taking daily-oral PrEP. Seven (43.8%) BLMSM reported ever having discontinued their PrEP use. Among those on LAI PrEP, nine BLMSM had switched from daily-oral PrEP, including one whose trajectory included switching from daily-oral to intermittent to LAI PrEP. All but one BLMSM who was currently taking daily-oral PrEP had never received LAI PrEP. The one current daily-oral consumer had switched back to daily-oral PrEP after experiencing negative side effects from the LAI.

**Table 1:** Participant demographics.

Variable	*N* (%)
Cisgender BLMSM Patients (*N* _= _16)	
Age	
18–29 years	10 (58.8)
30–39 years	6 (41.2)
Sexual orientation	
Gay	13 (81.3)
Bisexual	1 ( 6.2)
Pansexual	1 ( 6.2)
Queer	1 ( 6.2)
Race/ethnicity^a^	
Black or African American	11 (68.8)
Hispanic or Latino	6 (37.5)
Education Level	
Some college	5 (31.3)
Vocational/2-year degree	3 (18.8)
4-year college degree	6 (37.5)
Graduate school	2 (12.5)
Health Insurance	
Uninsured	4 (25.0)
Medicaid	3 (18.8)
Private Insurance	8 (50.0)
Refuse to answer	1 ( 6.2)
Financial Needs Met	
Just basic needs met	7 (43.8)
Needs met with a little extra	5 (31.3)
Lives comfortably	4 (25.0)
Housing Instability	
Yes	2 (12.5)
Clinical PrEP staff (*N* _= _7)	
Race/Ethnicity	
Non-Hispanic White	4 (57.1)
Black or African American	1 (14.3)
Hispanic or Latino	2 (28.6)
Gender Identity	
Cisgender male	3 (42.9)
Cisgender female	3 (42.9)
Gender expansive	1 (14.3)
Sexual orientation	
Heterosexual or straight	3 (42.9)
Gay, lesbian, or queer	4 (57.1)
Position	
Full-Time	7 (100.0)
HIV prevention service experience	
Less than 6 years	3 (42.9)
More than 6 years	4 (57.1)

a Rows are not mutually exclusive.

**Table 2:** Participant characteristics.

Patient	Age group	Race/ethnicity	Current PrEP modality	Any PrEP discontinuation	PrEP team staff	Clinical role
1	25–29	Hispanic/Latino	Daily-Oral	No	1	Medical
2	25–29	Hispanic/Latino	Long-Acting Injectable	No	2	Social services/Outreach
3	25–29	Black/African American	Long-Acting Injectable	Yes	3	Social services/Outreach
4	25–29	Black/African American	Long-Acting Injectable	Yes	4	Medical
5	18–24	Black/African American	Daily-Oral	No	5	Medical
6	30–39	Black/African American	Daily-Oral	Yes	6	Social services/Outreach
7	30–39	Hispanic/Latino	Daily-Oral	Yes	7	Social services/Outreach
8	25–29	Hispanic/Latino	Long-Acting Injectable	Yes		
9	25–29	Black/African American & Hispanic/Latino	Long-Acting Injectable	No		
10	18–24	Hispanic/Latino	Long-Acting Injectable	No		
11	25–29	Black/African American	Daily-Oral	Yes		
12	25–29	Black/African American	Long-Acting Injectable	No		
13	30–39	Black/African American & Multiracial	Daily-Oral	No		
14	30–39	Black/African American	Long-Acting Injectable	Yes		
15	30–39	Black/African American	Long-Acting Injectable	No		
16	30–39	Black/African American	Long-Acting Injectable	No		

PrEP staff (Table 1) included three full-time medical (42.9%) and four full-time social service/outreach providers (57.1%; Table 2). Four (57.1%) PrEP staff members self-reported as Non-Hispanic White, one (14.3%) as Non-Hispanic Black, and two (28.6%) as Hispanic/Latine. Three PrEP staff members self-identified as cisgender male (42.9%), three were cisgender female (42.9%), and one self-identified as gender-expansive (14.3%). Four (57.1%) PrEP staff members identified as gay, lesbian, or queer. Lastly, four (57.1%) PrEP staff members reported working in HIV prevention for at least six years.

### PrEP journeys

**Figure 1:**
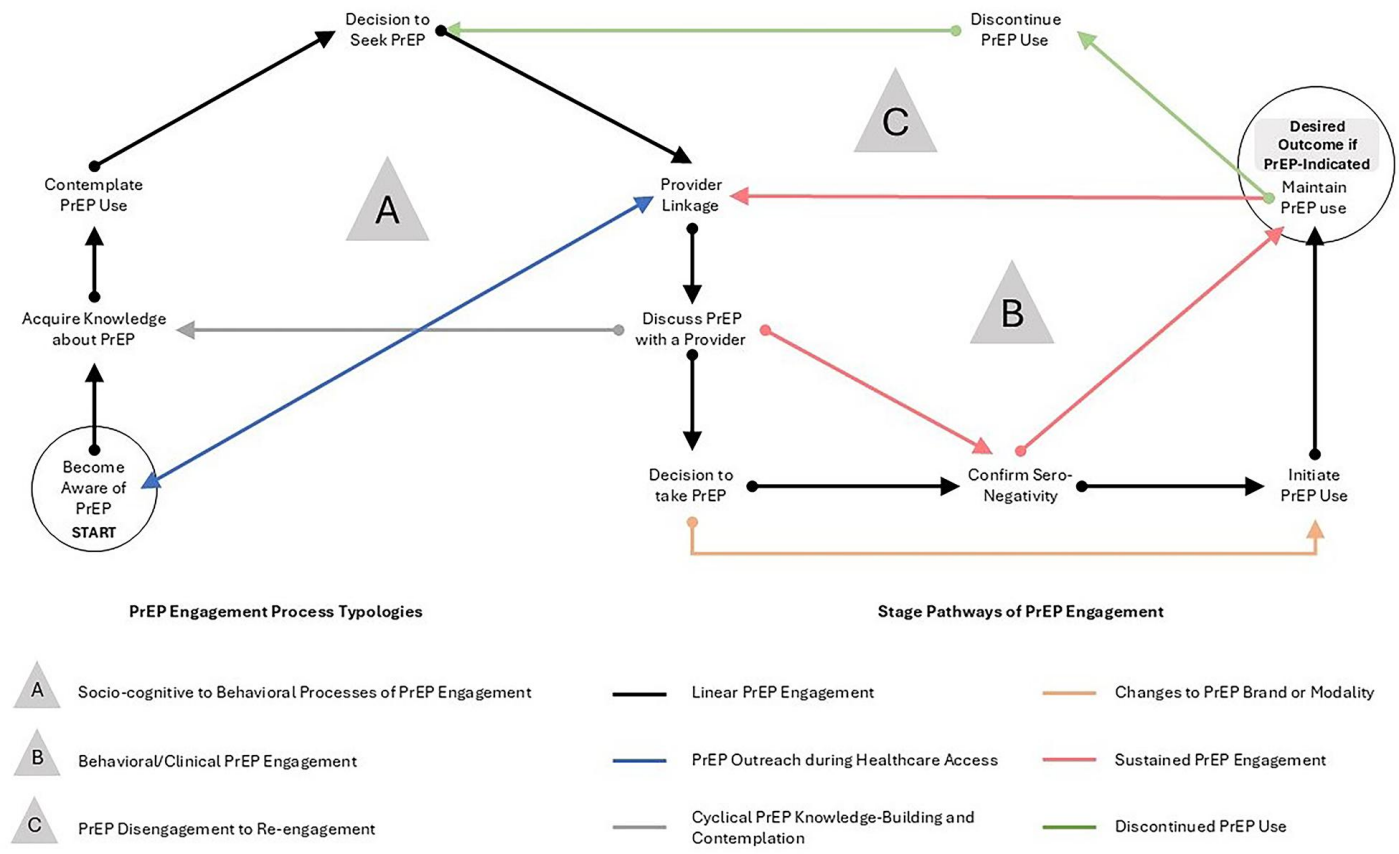
High-level patient journey map for PrEP-persistent cisgender Black and Latino men who have sex with men (BLMSM).

Figure 1 illustrates the high-level pathways of PrEP-engagement among BLMSM derived from journey mapping interviews, highlighting knowledge-building, decision-making, and behavioral transitions across the PrEP care continuum. The map depicts linear and cyclical movement across three PrEP process stages: (1) the early contemplative phases (Section A: Socio-cognitive transitions to behavioral PrEP engagement: from becoming aware of PrEP to discussing PrEP with a provider); (2) the active engagement phases (Section B: Behavioral and clinical PrEP engagement: from discussing PrEP with a provider to maintaining PrEP use); and (3) interruptions and care re-linkage (Section C: PrEP disengagement to re-engagement). Each section (A, B, and C) is depicted in detail in separate Figures. Below, we describe key patterns in BLMSM's PrEP journeys, leveraging representative quotes from BLMSM patients and PrEP staff (corresponding participant characteristics in Table 2), at each process stage.

### Socio-cognitive transitions to behavioral PrEP engagement

**Figure 2:**
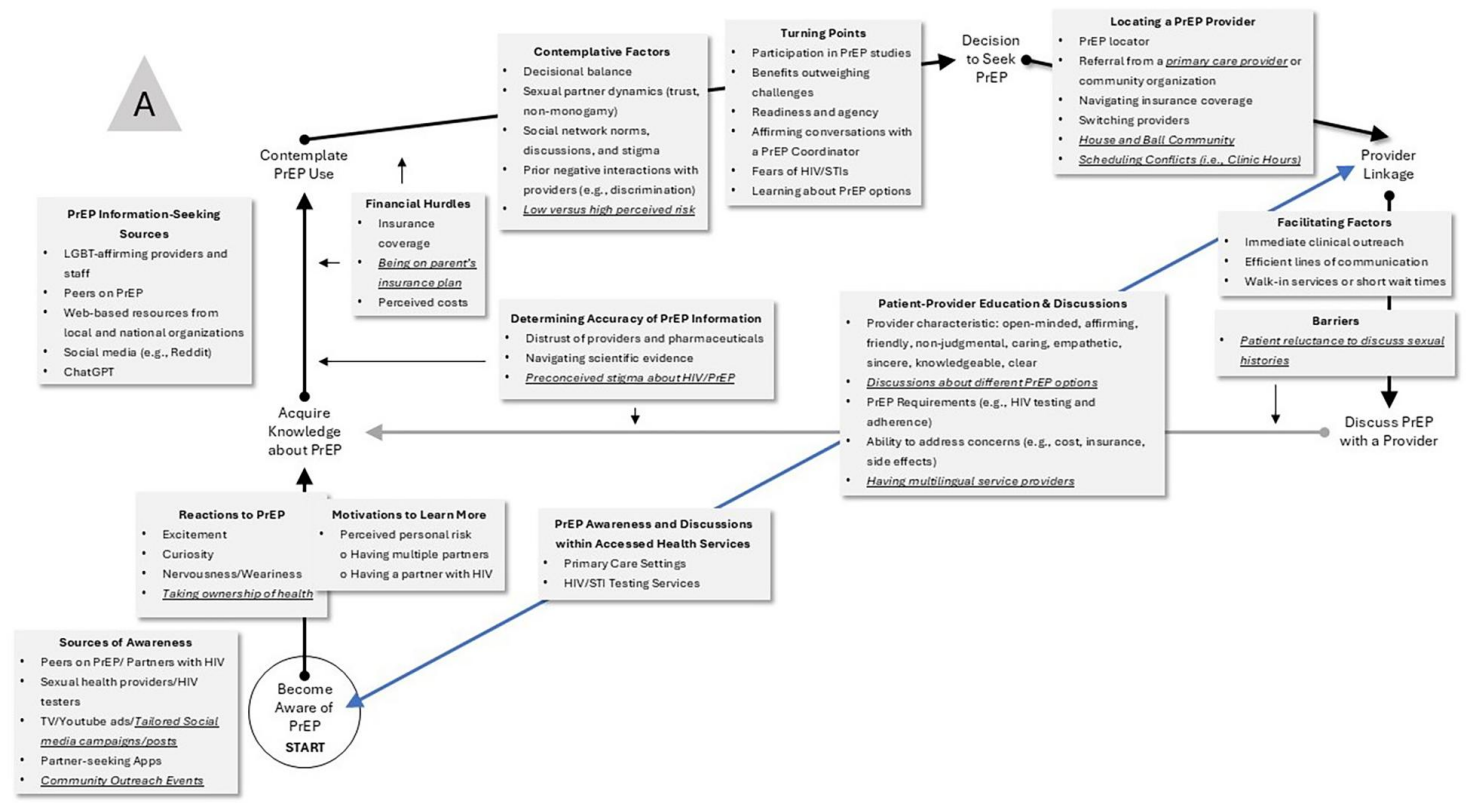
Social-cognitive transitions to behavioral PrEP engagement.

Figure 2 illustrates BLMSM and providers' perspectives on patients' socio-cognitive journeys as they move from first becoming aware of PrEP to actively seeking services and engaging in patient-provider discussions. This figure depicts a sequence of transitions, including initial awareness, knowledge acquisition, contemplation, PrEP service-seeking, and patient-provider discussions. As illustrated in the figure, participants described how resources such as peers, social media, and clinical encounters elicited interest and considerations about whether PrEP was right for them (Figure 1 – Section A and Figure 2).

#### Becoming aware of PrEP

BLMSM described their movement toward PrEP engagement as an iterative process shaped by evolving awareness, evaluation of personal risk, and appraisal of available resources. Patients and PrEP staff highlighted PrEP awareness often emerging either passively via sources such as peers, TV ads, and social media, or actively within the context of being educated about PrEP when already accessing services (e.g., primary care, HIV/STI testing). One patient described:

“…it was one of the gays [that] told me about [PrEP] at a happy hour or something when it was coming out. And then I think I talked to [my provider], but I didn’t have medical insurance at the time, so I was nota bout the cost of it.”—Patient 7

This quote highlighted that for some, PrEP awareness was accompanied by awareness of anticipated challenges to accessing the medication. When asked about helpful resources for raising PrEP awareness to BLMSM, one PrEP staff member opined:

“Probably the most helpful…social media, I think…and peer-to-peer advertisement…so somebody [who] follows a Black MSM that talks about being on PrEP is probably going to be one of the top ways that people get awareness that way…pharma companies for just advertisements for medication help, too. I’ve had patients come…asking for PrEP because they saw a commercial…but I think the advertisements…then word-of-mouth, I guess a little bit if their friends are on PrEP or their partners are on PrEP.”—PrEP Staff 1

Initial reactions to PrEP included cautious excitement, curiosity, and nervousness. When asked about how he reacted to becoming aware of PrEP, one participant recounted:

“I think I was like, yay to be able to do something to prevent HIV for myself just because in our community, condoms are not always [used]. And then I think the second reaction was, so I wonder what it does to the body.”—Patient 9

BLMSM's interest in learning more about PrEP arose upon reflecting about their own perceived risk, including whether they were having multiple sexual partners or a partner living with HIV.

#### PrEP knowledge acquisition

Participants described several in-person and online resources that supported learning about PrEP and the clinical and behavioral requirements for maximizing its benefits. In-person resources typically included healthcare providers, PrEP-specific staff (e.g., PrEP coordinators), and peers who were currently on PrEP. BLMSM largely endorsed their providers as their most trusted source for accurate PrEP information, particularly when providers were perceived as non-judgmental, sexuality-affirming, empathetic, and knowledgeable. One participant discussed:

“Having that one-on-one with my PCP and actually sitting down and having a conversation with me about it, tell me the side effects, the risk, as well as the beneficial parts…the pros, how they do outweigh the cons. I guess it made it…very much easier to understand.”—Patient 5

Another participant relayed:

“I told [my provider] I was a ho. Once I felt comfortable and I was able to talk with them, it was easy for me to have [discuss my sexual history], but I was terrified…he made me feel comfortable, safe. So it allowed me to ask for [PrEP] right away. Honestly, if it wasn’t for the fact that he made me feel safe and comfortable…I probably wouldn’t have approached that conversation with any other [provider].”—Patient 13

Positive provider discussions helped allay underlying mistrust of healthcare systems and the pharmaceutical industry. Additionally, online PrEP resources identified by participants included websites from healthcare institutions (e.g., clinics, PrEP manufacturers, CDC), social media (e.g., PrEP subreddits, partner-seeking apps), and AI chatbots (e.g., ChatGPT). As one participant described:

“It is on Grindr, you jump on Badoo, you jump on all these other sites [like] Sniffies…Adam4Adam…it's there. So that fact that it's just one click and that's all it takes, you’re exposed to PrEP. I think that's the best thing ever…It's there in the conversation app…They [are] able to have that conversation and now you are arming that person with the beginning building blocks to move forward in a safe-sex positive life.”—Patient 13

#### Contemplating PrEP use

BLMSM described weighing the information they learned about PrEP largely considering their socioeconomic situation and their interpersonal relationships. Many participants highlighted the expensive cost of PrEP medications, an especially salient factor among uninsured participants. One participant said:

“The biggest worry for me was the side effects how it impacts my day-to-day and impacts to my job if there were any [side effects]. And then…[how taking PrEP] impacts to my finances, if there was any out-of-pocket expense, copays, what have you.”—Patient 16

Financial concerns, among other socioeconomic concerns, were echoed by PrEP staff. One staff member argued:

“I would say cost or insurance…is probably number one. Younger patients may be concerned about where they’re living…if they live [with family] or their insurance is under a parent's insurance…I think housing stability…if they’re not living at home and they’re struggling, they’re experiencing homelessness or bouncing between shelters and group homes, being able to manage either getting to appointments or picking up a prescription and keeping the bottle safe and taking a pill every day, and sticking with that regimen is probably another big barrier…”—PrEP Staff 1

As alluded to in the quote above, some participants discussed perceived challenges related to being a dependent on their parents' health insurance coverage and fearing that their parents may come to find out or make assumptions (e.g., sexuality, HIV status) about their PrEP use. Additionally, PrEP-related decision balance was connected to participants' sexual activity and perceived HIV risk. Many BLMSM reflected on the reassurance PrEP provided in the context of being in non-monogamous partnerships and having distrust toward casual partners' sexual histories. Perceptions of taking PrEP were also shaped and reinforced by normative discussions within peer networks, highlighting widespread use and endorsement among friend circles. For example, one BLMSM participant relayed:

“It was my gay best friend…he had been on PrEP. He had started it maybe a couple months before I did, and he said to me, ‘Yeah, I feel fine. I take it every day’…so once he told me that…I decided, alright, cool. I can really do this.”—Patient 3

Similarly, one PrEP staff member recounted:

“I’ve had patients who say that their friends refer them here, [they] say…‘I saw my partner taking PrEP and I talked to them about it and they taught me a lot.’ So they’ll come into the appointment knowing everything and I barely have to do any education…having that support is really helpful.”—PrEP Staff 3

Final decisions to seek PrEP emerged as participants perceived benefits to outweigh challenges, felt ready and capable of engaging in PrEP care, had affirming conversations with PrEP staff (including learning about different PrEP options), and reflected on fears of acquiring HIV or a bacterial STI. One participant described:

“I was having more sex…[being] more explorative with people. I had been in a relationship for a little while, so just coming out of that was kind of like…how do I mitigate the risk in such a way that allows me to have fun without feeling all this anxiety about illnesses afterwards?”—Patient 8

#### Seeking PrEP services

Once they had decided to begin PrEP, BLMSM described their processes for identifying an appropriate provider. BLMSM not previously linked to healthcare services discussed several strategies for identifying a local PrEP provider, e.g., online resources, such as Google searches and PrEP locators, often accessed through partner-seeking apps (e.g., Grindr). Once linked to PrEP services, having immediate outreach by clinical staff for care coordination, leveraging efficient lines of communication (e.g., via phone call, texting, or patient portal messages), and having walk-in services with short wait times all supported access to PrEP providers. Though no BLMSM participants discussed challenges getting PrEP appointments, PrEP staff indicated persistent scheduling conflicts with limited available service hours as barriers to appointment planning. PrEP staff, nevertheless, discussed several strategies for assisting BLMSM overcome linkage barriers including PrEP assistance programs. One staff member elaborated:

“Getting them in a patient assistance program and saying like, ‘Hey, we can get you this medicine for free. We have the ability to cover all your lab costs. You don’t have to pay anything when you come in for a visit’…the cost aspect…[being] able to see uninsured patients and able to provide medicine….that's probably the number one way…And then the tele-PrEP for oral PrEP patients is another one.”—PrEP Staff 1

Among those already connected to healthcare or community-based organizations, many were referred to PrEP staff by primary care providers and some discussed actively searching for new providers after interacting with non-affirming providers. PrEP staff highlighted how some BLMSM had a reluctance to discuss sexual health and histories, inhibiting opportunities for them to discuss PrEP as an HIV prevention option with their patients.

#### Patient-provider PrEP discussions

BLMSM largely endorsed provider education and discussions at FIGHT as fundamental to learning about and initiating PrEP. Overall, patients viewed their providers as highly knowledgeable about PrEP. Beyond the personable characteristics (e.g., sincere, empathetic, non-judgmental) of all PrEP team staff, many BLMSM appreciated their provider explaining the different options for taking PrEP, the requirements for use, and the ability to discuss PrEP's benefits and address concerns in non-technical ways. One participant recounted:

“I definitely relied on my providers…[they] were also very honest about where the area of science was not clear…I think that that's something that really grounded me in my decision…I remember having this long conversation about those edge cases where it's like…the people were on PrEP, but then they also got HIV…[I asked], ‘Why do you think that happened?’…Being able to have those kinds of conversations…I think that made me really appreciate…the information.”—Patient 2

PrEP staff described discussing PrEP with all sexually active patients. Some BLMSM discussed having providers who were multilingual and shared similar backgrounds or lived experiences to support positive PrEP discussions. As one staff member described the role of having a multilingual PrEP coordinator:

“If the patient speaks Spanish as their first language, he’ll come up and pretty much have all those conversations with the patient…it's not just for language purposes…I think the nuances of getting to use innuendo are kind of lost in medical settings when you have to speak in a different language…so that's pretty much how we navigate that.”—PrEP Staff 2

When discussing how they addressed healthcare mistrust, another staff member relayed:

“I’m Black presenting, it lands differently than if someone else were to say it…I say, ‘Hey, look, the healthcare system is messed up. There's a lot of things that have happened that are really bad…and are disproportionately harming Black and Latino people. However, this option that we have, HIV PrEP is something that is a really great option to [protect] yourself. And if it wasn’t such a good option, we wouldn’t see such a high uptake in White MSM, especially affluent White MSM…I mean it sucks you have to look at it that way, but that should help give you a little bit of peace of mind that okay, there must be some good to it.’”—PrEP Staff 3

### Behavioral and clinical PrEP engagement

**Figure 3:**
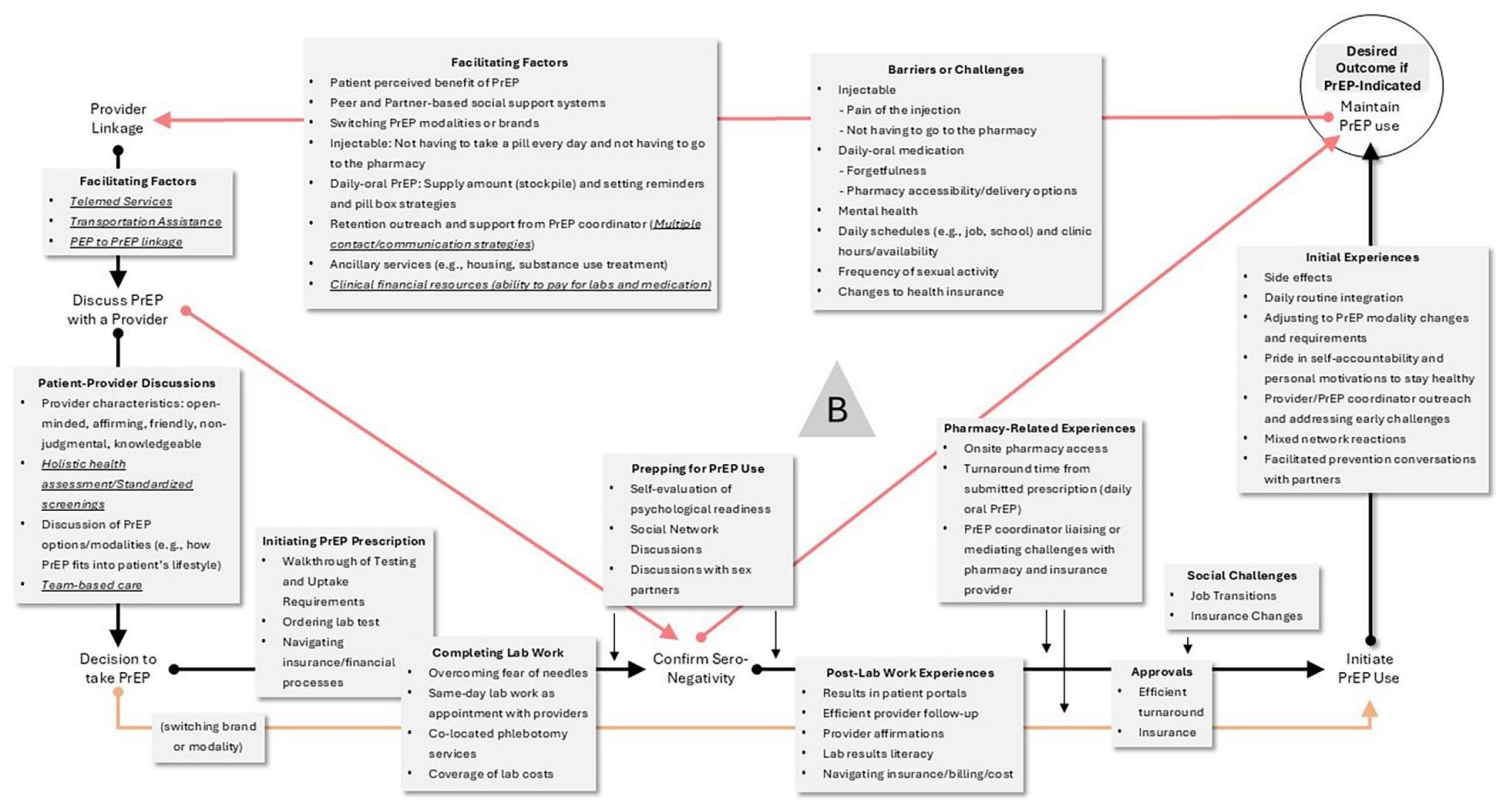
Behavioral and clinical PrEP engagement.

Figure 3 illustrates in detail BLMSM's behavioral and clinical journey as they moved toward initiating and sustaining engagement in PrEP care. This figure depicts an often-cyclical sequence of behavioral and clinical touchpoints, including preparations to start PrEP, completing all the necessary requirements (e.g., confirming sero-negativity and navigating PrEP-related costs), starting and integrating PrEP into daily routines, adhering to dosing, and attending routine monitoring visits (Figure 1 – Section A and Figure 3).

#### Patient-provider PrEP discussions

Behavioral and clinical engagement processes began with dynamic patient-provider discussions, given that PrEP requires a medical prescription. In contrast to the provider discussions described in the socio-cognitive phase, discussions in this phase occurred *after* a patient had decided to seek PrEP (as opposed to reaching out to a provider to learn about PrEP). Within these discussions, BLMSM consistently described providers' critical role in maintaining open, affirming, and non-judgmental conversations when educating and emphasizing the importance of routine HIV testing, taking PrEP as prescribed, and conducting individualized prevention counseling. At this stage, patient-provider discussions were especially critical to maximize BLMSM's capacity to prepare to start their PrEP regimen. When asked about provider discussions while seeking PrEP, one participant shared:

“I think…the ability to ask all my questions…having the opportunity to engage in that conversation with someone else who was very sex-positive and very accepting…it is rare…I was grateful for a space to ask…all my weird questions [about PrEP].”—Patient 4

Patients described how providers' (medical and social service-related) patient-centered practices, such as facilitating shared decision-making around minimizing anticipated challenges that PrEP could pose to their daily routines. For BLMSM who started on Truvada, many provider discussions over the PrEP journey involved becoming aware of new PrEP options, like Descovy and the bimonthly LAI, as they became available. One participant said:

“When I first started, the only thing that was available was the daily medication…my options were between Descovy and Truvada…I had heard weird things about Truvada, so I chose Descovy and Descovy was newer…Truvada reminded me of a fish oil pill, like a big vitamin, I didn’t want to take that every single day.”—Patient 4

#### Preparing to initiate PrEP

After making a final decision to start PrEP (or switch brand/modality), BLMSM described barriers and facilitators to confirming their negative sero-status. Several participants described challenges, including fear of needles (for blood draws and considering use of LAI PrEP) and persistent concerns about navigating cost and insurance. Fear of needles was frequently deemed surmountable and many BLMSM appreciated barrier-mitigating support via the availability of same-day, co-located phlebotomy services at their PrEP appointments and FIGHT's ability to assist covering lab costs. One participants recounted:

“The first time, I definitely was nervous…I’m not a big fan of needles…also…how many tubes it actually took [was] kind of nerve-wracking when you look over and it's just a bunch of tubes sitting there…I guess I kind of just got used to it the longer I was on the medication and coming back for labs repeatedly.”—Patient 11

The journey between BLMSM confirming their sero-negativity and initiating PrEP use frequently encompassed several multilevel processes. For those new to PrEP, moving toward PrEP initiation involved reflecting on their readiness to engage, especially for those who started on the daily-oral regimen. Several participants recounted mixed (i.e., some supportive, some stigmatizing) discussions with their peer networks and sex partners about getting ready to start taking PrEP. The few BLMSM who experienced negative reactions described being undeterred based on valuing their autonomy and health. One patient described:

“My ex-boyfriend, when I first told him about it…it was a question of, well why [do] I need to get on PrEP if we’re together, exclusive. I was like, ‘because life happens and it only takes one time.’ You’re protecting yourself…And he still wouldn’t get on it and I couldn’t understand that, but I was like…I know myself and I know that I get tested often…even after every partner just so I can be safe…You can only control yourself. So why not project yourself?”—Patient 4

Regarding clinical and health system processes, BLMSM discussed their providers' thorough, efficient, and affirming communication of lab results in-person and via patient portal systems. Provider follow-up supported patient literacy and understanding of screened biomarkers. For example, one participant shared:

“For the first few times we got the blood work, they went over it in detail…I was kind of better able to gauge my body's reaction to it…anytime I got the blood work…I’d be like, ‘Hey, this number seems altered compared to the last one…What would cause that?’…they would hop on the phone…explain it to me… it was more of just that open communication where I could ask questions and they would get back to me…usually at the same day.”—Patient 7

#### Navigating pharmacy and insurance challenges

Many participants lamented common and complex challenges regarding their interactions with pharmacies and insurance approvals. This issue was particularly salient for men who transitioned jobs and experienced health insurance status and coverage changes. Nevertheless, BLMSM emphasized the critical supportive role that FIGHT's PrEP coordinators played in liaising and mediating challenges between pharmacies and health insurance companies. One participant described:

“[The PrEP coordinator] was just the person, the liaison, the person on the phone…with the health insurance and stuff. A couple of times I would’ve came to using the copay card system…But when it was through my own pharmacy benefits alone, they were always creative about options I could do.”—Patient 4

When discussing a pharmaceutical program that provides copay assistance, a clinical PrEP team staff member described it as a program for patients who were uninsured or low-income, stating, “*It's a wonderful program, we get Descovy, Truvada just with an application, it can be shipped to [the patients’] houses.”* For patients with insurance, he further discussed:

“Whenever a patient has insurance, a good one, gets prescribed Descovy, gets immediately rejected…so we have to appeal several times. They ask us to put them on Truvada first on the generic side and then to go to Descovy. So it takes six months for some patients who have insurance and good insurance and for us to navigate and to get their prescription.”—PrEP Staff 6

#### Initiating PrEP use

After starting PrEP, BLMSM described diverse experiences integrating PrEP into their daily routine. One of the most common challenges for daily-oral PrEP users was remembering to take their PrEP medications every day. Participants described a number of strategies, many of which were recommended by PrEP clinic staff, to support PrEP adoption, including setting alarms, taking medications during consistently convenient times of day, and traveling with their PrEP medications during busy days. For example, one participant shared:

“Taking the pill once a day, it's hard to remember…they were offering me, I think I could get a text message a day or an email or some kind of reminding system…to help remind me to take the pill. I didn’t really take them up on that because I would just find it annoying…so I set an alarm for the first few months, but we kind of just discussed that.”—Patient 7

Another common challenge was navigating side effects when their bodies were adjusting to the medication. This was especially salient for individuals who switched from daily-oral medications to the bimonthly LAI. Ultimately, BLMSM described starting PrEP as fostering a sense of pride in self-accountability and facilitated conversations about HIV prevention with new partners. As one participant highlighted:

“[PrEP] made me feel…I guess you could say it made me feel a little bit more responsible about myself. I did feel comforted in the fact that now I was required to get tested every month, so that way I’m not living in doubt or having to make phone calls that I felt were embarrassing to make to a random clinic or something.”—Patient 1

#### PrEP persistence

Barriers and challenges to BLMSM's PrEP persistence included injection-site pain from LAI, continued forgetfulness with daily-oral medication, and challenges accessing pharmacies. Mental health concerns, busy schedules, limited clinic hours, infrequent sexual activity, and changes in health insurance further hindered consistent use. Despite these challenges, BLMSM described how their own internal motivations and clinical supports helped to remain engaged in their PrEP Care. Clinical support included ongoing discussions about PrEP options with active retention outreach from PrEP coordinators. Access to peer and partner support networks, referral to ancillary services, and financial resources for labs and medication also helped to mitigate economic and psychosocial barriers. As one participant relayed:

“They have [PrEP coordinators] to help you with the lab work, the medication itself, the different timeframes that you need to take the medication in. They’re always reaching out to you to tell you, ‘you need to schedule it and these are your dates…’ if you need any financial assistance, they somehow find a way to get [it] for you for anything that's not covered.”—Patient 15

Consistent education (including raising awareness or revisiting alternative PrEP options), reassurance, and provider commitment are central to overcoming barriers and supporting long-term PrEP engagement. This was reflected when one clinical PrEP staff member conveyed:

“What we can do is be persistent about offering solutions…if I’m in the office with someone and they’re like, I’m really having a hard time figuring out how this is going to work, my favorite thing to do is to have a full plan of like…here's what I’m thinking…we’ll try having this list of options and worst-case scenarios…I have found that patients…there is relief of like…we have plan B and C.”—PrEP Staff 7

Lastly, both BLMSM and staff described that when necessary, FIGHT has offered tele-med appointments, walk-in appointments, and transportation assistance to ensure sustained PrEP care when BLMSM patients had scheduling or travel-related barriers.

### PrEP disengagement to re-engagement

**Figure 4:**
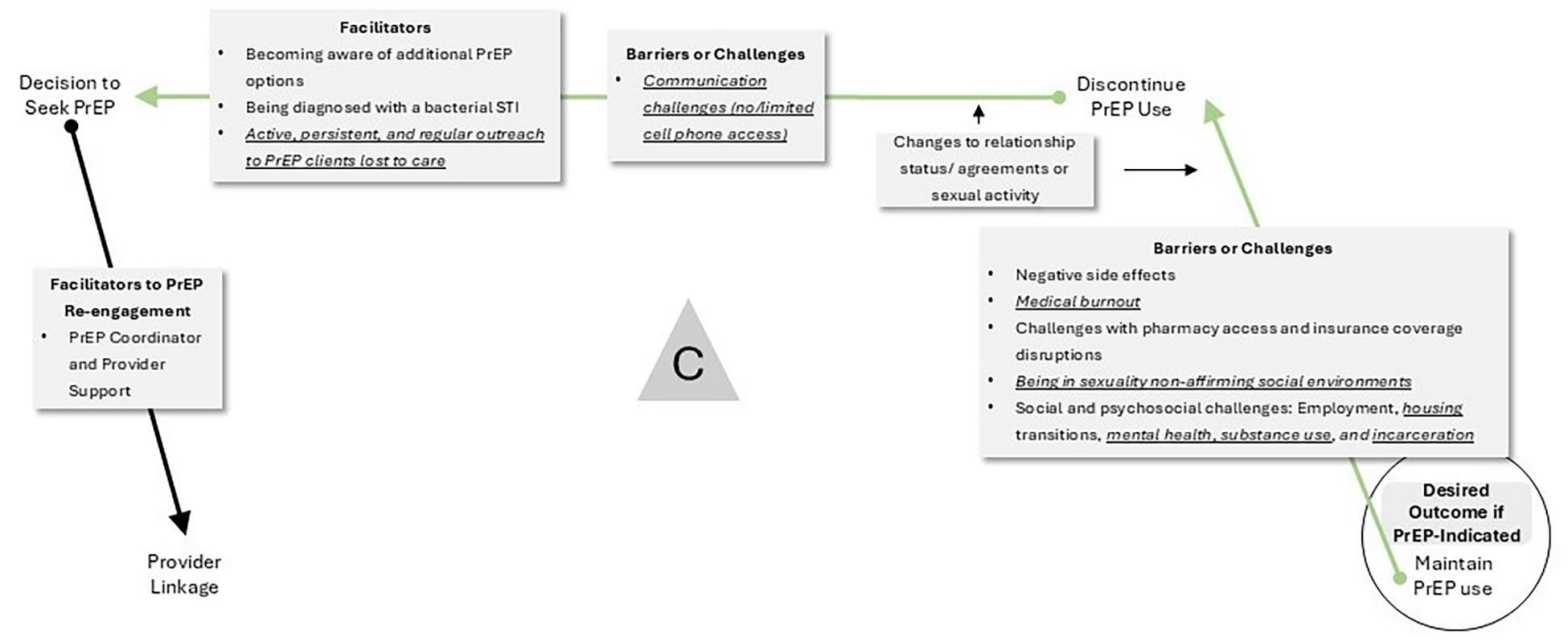
PrEP discontinuation to re-engagement.

Figure 4 illustrates the journey BLMSM and PrEP staff described when patients transitioned into periods of discontinuation (both intentional and unintentional) and, for some, re-engagement in PrEP care. Seven of the 16 (43.8%) BLMSM patients reported and provided insights into their experiences of PrEP discontinuation or interruptions, re-engagement, and eventual return to sustained PrEP. This figure depicts the dynamic processes in which structural conditions, interpersonal relationships, PrEP experiences, and evolving risk appraisals shaped how BLMSM re-engaged with PrEP services (Figure 1 – Section C and Figure 4).

#### Reasons for PrEP discontinuation

BLMSM described different reasons for interruptions in PrEP engagement, including shifts in relationship status (e.g., starting a sexually monogamous relationship) and perceived sexual risk. As one participant indicated:

“I was…on PrEP for, I want to say at least about two years because I was being promiscuous for a year… then I started dating someone who was actually HIV-positive…it was actually very helpful, that I was on PrEP, so I stayed on PrEP with that… then we’d broken up and…when I started a new relationship the following years…maybe three years, I stopped taking it because I didn’t need to any longer…at that time we were just in a monogamous relationship.”—Patient 14

Participants described barriers at this stage to include persisting negative side effects, stress, burnout, and fatigue from consistently having to coordinate and undergo all the logistical requirements necessary to sustain PrEP use, scheduling conflicts with clinic hours, and ongoing challenges navigating pharmacy access and approvals. One participant lamented:

“At a certain point I would quit and then one and a half, two-year frame when I was taking the Descovy, I was just tired of dealing with the nonsense at the pharmacy…I was just tired of the nonsense and at that time, I wasn’t really sexually active so I wasn’t too worried…[then] they told me about the Apretude becoming available in the near future…knowing that this was every two months or so…I’m like, I don’t have to deal with the nonsense at the pharmacy.”—Participant 4

Aligned with this participant, one clinical PrEP staff member reflected:

“Part of me wonders if medical burnout is part of it. I think because the constantly having to get labs, constantly having to come to appointments to talk about sexual behaviors and activities and just all the things. I think medical burnout is real. I think medication adherence or the frequency of taking medication we can burn out from.”—PrEP Staff 5

BLMSM and PrEP staff highlighted ongoing structural and social challenges, such as job or housing transitions, insurance disruptions, incarceration, and non-affirming social environments, coupled with mental health and substance use concerns, that further complicated efforts for BLMSM and the clinic to sustain PrEP engagement. One patient elaborated:

“I was going through my job transitions and then I had just turned 26, so I was off my parents’ insurance and then I had just got a new job… my old insurance had ended, but I couldn’t start my new one because I was still on my probationary period…[FIGHT] did end up giving me a couple of free bottles for a couple of weeks; however, I don’t think I started, I didn’t take them because obviously life was being life.”—Patient 3

When asked about addressing socioeconomic barriers, a PrEP staff member described:

“We had a patient who…was really struggling to come to clinic visits and was having transportation barriers…we were trying to mail [transportation] passes, but his housing was unstable and wasn’t really in one place…I think he was almost at the end of his window or something for his Apretude…so we were able to send him an Uber to bring him to the clinic from, I think his workplace is where he came from.”—PrEP Staff 2

[Re-engaging PrEP…suggested sub-header]

#### PrEP re-engagement

Despite these barriers, several facilitating factors supported movement back toward PrEP use (see Figure 4). Two facilitators from that seemed to be particularly important in this phase were knowledge about PrEP modalities and men's ongoing assessments of their own vulnerability to HIV. Many BLMSM sought to re-engage in PrEP care when they became newly aware of additional PrEP options, such as the LAI. Others reconsidered PrEP after a bacterial STI diagnosis, facilitating renewed perceptions of HIV risk. For example, one participant recounted:

“I had tested positive for gonorrhea and I was just like, oh gosh, out of 26 years, I’ve never caught anything and I’m fucking terrified…I was like, I need to get back on [PrEP]… I know PrEP is not for that, but I really felt safer with it at that time…if you can give me this, you can give me something else, and that's all I kept thinking about.”—Patient 3

As noted by both BLMSM and PrEP staff, active, persistent clinical outreach from PrEP coordinators to patients who had fallen out of care was described as being especially influential. PrEP team staff emphasized the dedicated role of PrEP coordinators looking out for patients, providing reminders via text messages, making sure they were able to access their clinic visits, and quickly re-scheduling missed appointments. Financial navigation support helped participants manage medication and visit costs, reducing a key barrier to re-initiation. Coordinated efforts by PrEP coordinators and providers to maintain or restore communication, even when clients had limited or no cell phone access, were critical in re-establishing contact and rebuilding trust. When asked about reaching back out to their PrEP provider, one participant said:

“I’ve always had the same provider. I have not had the same PrEP coordinator, and the PrEP coordinator is always the one that reaches out, gets me back on track…[They] reach out and make sure…when it comes to my Apretude to make sure my injectable's there and delivered on time and that I’m receiving it in the appropriate window.”—Participant 4

PrEP staff described re-engaging BLMSM to PrEP was a major undertaking but remained undeterred in implementing consistent retention and outreach strategies. One staff member highlighted:

“Having dedicated PrEP [coordinators] in each clinic and an actual program in place…So we meet once a week and we discuss big picture…program management and outreach…we also do numbers and we talk about how many active PrEP patients we have, how many are on oral PrEP, how many on injectable PrEP, how many are lost to care?…How many patients are overdue? We have a deactivated list that we’ll reach out to every once in a while. So patients that have said, I don’t want to be on PrEP anymore…we say, ‘Can we call you in a month though and ask you about PrEP again?’ And if they say yes, we’ll call them in a month…So really having a dedicated team or a dedicated person at least that can keep track of patients, because keeping patients in care is definitely a barrier for us and certainly a barrier for patients.”—PrEP Staff 1

When discussing strategies to support re-engagement among BLMSM who express interest, one staff member described:

“[We say] that we can see them whenever they can come in. So not saying, we’re going to make an appointment for you in three weeks, but if contact is made, then being able to say… “Come in tomorrow and we’ll see you.” And we have walk-in slots that we save for people, and we tell people, not just for re-establish, but for PrEP starts, we’ll see new patients as a walk-in… we’ll see new patients as a walk-in for a PrEP start or for needing for PEP after exposure. And I would include in that reengaged populations.”—PrEP Staff 4

The pathway ultimately reconnects individuals to provider linkage, where tailored counseling, updated information on available PrEP modalities, and practical problem solving around structural barriers support a return to and maintenance of PrEP use when clinically indicated.

## Discussion

This study provides several important contributions to health services research aiming to support PrEP engagement among cisgender BLMSM. First, our study is among the first to apply qualitative journey mapping to BLMSM's experiences receiving PrEP within an FQHC. We generated a detailed visual and narrative account of how socio-cognitive, behavioral, and re-engagement processes unfold over time in a low-barrier clinical context, with particular attention to participants currently sustaining PrEP engagement after encountering barriers and treatment interruptions. Among our primary findings, we observed that for some BLMSM, PrEP engagement may exhibit linear engagement along the PrEP care continuum. For others, PrEP journey maps surfaced multiple pathways from PrEP awareness to maintenance that include cycles of socio-cognitive engagement, temporary pauses, modality switches, and cycles of disengagement. These maps showed how BLMSM move back and forth across stages in response to shifting life circumstances, structural constraints, and evolving perceptions of HIV risk. For several participants, becoming aware of and being approved for the bimonthly LAI served as a critical turning point for initiating PrEP, being retained, and re-engaging in PrEP care.

At a conceptual level, our findings extend prevailing PrEP care continuum and journey mapping frameworks by advancing a patient-centered, resilience-informed model grounded in leveraging internal assets and supportive resources via shared decision-making with PrEP staff when BLMSM are faced with multilevel social and economic challenges. Similar to prior work, many BLMSM described embracing PrEP as taking control of one's health, providing self-reassurance about preventing HIV, not only for themselves but for their community, and allowing themselves to have sexual intercourse with multiple partners with less anxiety (37). Alongside positive clinic interactions, these beliefs and values facilitated PrEP engagement despite logistical requirements (e.g., fear of needles when accessing phlebotomy services or initiating the bimonthly LAI) and stigmatizing reactions from social networks.

BLMSM's experiences frequently converged with PrEP staff perspectives on clinic level supportive mechanisms. Patients and staff highlighted having dedicated PrEP coordinators, co-located and integrated services, proactive financial navigation, and flexible visit modalities as being able to transform common barriers such as insurance navigation, pharmacy challenges, and unstable housing into opportunities for retention and re-initiation. Echoing patterns documented in prior studies (17, 51), the most prominent and recurring barriers described by BLMSM and PrEP staff across all stages of the PrEP care continuum were perceived cost and insurance coverage. BLMSM and PrEP team members described patients’ confusion regarding how insurance works, uncertainty about eligibility and out-of-pocket responsibilities, and limited awareness of financial assistance programs that can cover appointments, phlebotomy, and PrEP medications.

These challenges generated frustration, and for some, burnout or fatigue related to coordinating, engaging, and interacting with all the necessary touchpoints (e.g., phlebotomy services, pharmacies) required for sustaining PrEP use. Building on emerging, but poorly understood, interest in documenting PrEP fatigue as a growing concern among PrEP users, our findings illustrate how cumulative hassles can erode motivation to remain engaged in PrEP services (52). These experiences align with studies of BLMSM that identify high or unpredictable costs, lapses in insurance, and difficulties navigating complex coverage requirements as common reasons for not starting PrEP or for discontinuing use despite ongoing HIV risk (53). In the current study, PrEP coordinators emerged as critical intermediaries who help offset these entrenched structural barriers by explaining benefits, securing patient assistance, appealing denials, and coordinating with pharmacies so that coverage gaps and co-pay burdens do not automatically result in PrEP interruption. Together, these findings reinforce prior evidence that financial and insurance systems are central drivers of PrEP inequities for BLMSM and highlight the importance of dedicated navigation roles within safety net settings to translate fragmented payer landscapes into feasible pathways for sustained PrEP engagement (6, 54, 55).

Building off active clinical support systems, BLMSM and staff members described how the PrEP team made themselves consistently available to patients for concrete, day-to-day problem solving. PrEP team members invited questions about lab results, side effects, and changes to sexual behaviors or practices, and translated clinical information into clear guidance that participants could apply in their routines. Rather than relying on one-time instructions, staff partnered with BLMSM to brainstorm adherence strategies, such as aligning pills or injection visits with existing schedules, planning around anticipated pharmacy or insurance delays, and revisiting these plans as factors like work or mental health shifted. This collaborative dimension empowered BLMSM to leverage feasible, agentic solutions that fit within daily lives.

Comprehensive communication strategies leveraged by PrEP staff such as patient portal messaging, texting, phone calls, and tele-med served as an adjunctive intervention toward strengthening BLMSM's PrEP persistence (56). Persistent communication strategies exemplified PrEP staff's commitment to supporting outreach and motivating sustained PrEP use, especially in the context of PrEP discontinuation and re-engagement. Aligned with prior studies emphasizing the importance of effective patient-provider PrEP communications, BLMSM described discussions and the communication styles of PrEP staff to be affirming, culturally attuned, informative, nonjudgmental, and provided in a non-technical manner (57, 58). This spirit of accessibility and collaboration signaled that PrEP engagement was a shared process, which helped patients view challenges as issues to work through with the team instead of as individual shortcomings, thereby supporting persistence across the continuum of care.

### Health practice and policy recommendations

Though PrEP awareness remains high among BLMSM, building awareness of alternative modalities as they emerge must be prioritized. Building awareness of alternative PrEP formulations should be paired with patient-provider discussions around how available options fit into BLMSM's lives over time and with guidelines pertaining to processes for switching brands or modalities (59). This is especially critical for BLMSM who experience PrEP fatigue or who are interested PrEP re-engagement after discontinuation and renews conversations about episodic PrEP and if appropriate, post-exposure prophylaxis (60–62). Clinical stakeholder training opportunities can ensure that discussions about PrEP options are presented in accessible terms and support culturally-tailored outreach that highlights real-world, peer-informed experiences across the PrEP care continuum (63). These discussions offer critical opportunities to preempt commonly perceived barriers. Training opportunities could include building clinical providers' comprehensive understanding of what resilience in PrEP uptake and persistence looks like in their patient populations, including which social, financial, and organizational resources patients use to mitigate barriers and the specific actions that clinics and clinical staff can take to support resilience processes.

Mapping resilience-supportive assets and resources can inform where to concentrate critical investments in PrEP infrastructure, including having and securing dedicated PrEP coordinators. PrEP coordinators serve as consistent and trusted points of contact and outreach for (1) navigating insurance, financial assistance programs, and pharmacies, (2) troubleshooting adherence challenges, coordinating transportation, scheduling, and other retention efforts, and (3) linking patients to ancillary services. As the most consistently identified barrier among BLMSM and PrEP team members in our study, efforts to expand BLMSM's awareness and uptake of financial assistance and contingency programs are critically needed. Particularly for individuals experiencing social transitions (e.g., employment and housing) characterized by socioeconomic instability, these programs can offset visit-, phlebotomy-, and medication-related costs. In the context of potential and ongoing changes to Affordable Care Act marketplace and Medicaid eligibility redeterminations that threaten coverage continuity, strengthening insurance navigation and benefits counseling within PrEP programs will be essential for keeping BLMSM connected to low- or no-cost PrEP. Integrating brief financial counseling (e.g., to support literacy of health insurance coverage) into PrEP education, initiation, and maintenance visits and providing clear written or digital materials can help patients identify and activate these resources before potential disruptions occur; however, given historical reliance, sustaining these roles and capacities will require continued federal and state-level investment (64).

### Limitations

This study has several limitations. The qualitative sample was small and recruited from a single FQHC, which limits the generalizability of our findings to BLMSM in other geographic or care settings. Additionally, qualitative interviews were conducted via Zoom to reduce travel burden, accommodate varied schedules, and support participation among individuals who might otherwise be unable to attend in-person visits. The virtual format may have constrained interviewers' ability to observe some nonverbal cues and to build interpersonal trust in the ways that can occur in shared physical spaces. Some participants may have felt less willing to disclose highly sensitive information due to concerns about privacy in their own environments (65). At the same time, interviewing online appeared to enhance feasibility and, for many, privacy and comfort by allowing them to join from a self-selected setting when discussing sexual health and PrEP.

Participants were asked to reconstruct PrEP journeys that, for many, spanned multiple years; therefore, similar to other journey mapping studies, retrospectively obtained narratives may be affected by recall bias, particularly around timing, the salience of specific and forgotten barriers, and the sequencing of decisions (66, 67). Though we sampled participants who had been PrEP persistent through at least two consecutive PrEP visits, we did not screen nor consider the duration of PrEP uptake and persistence among participants. Duration of PrEP use may have additional implications for barrier exposure and cultivating the necessary internal and external resources to support sustained engagement. Self-selection is also likely, as BLMSM who agreed to participate may have had more positive relationships with the clinic, greater comfort discussing sexual health, or stronger investment in PrEP than those who declined. In addition, the focus on individuals currently engaged in PrEP care means that the BLMSM who are PrEP-naïve or who have discontinued and not re-engaged are not represented. Socio-cognitive and behavioral processes involved in identifying sources and strategies to overcome barriers and perceived capacity may differ in these cases. Though BLMSM in our study self-reported to be more socioeconomically stable, PrEP staff's perspectives helped provide insight into observed and persistent challenges among individuals with limited socioeconomic resources.

### Future research

Despite in-depth insights into resilience processes in accessing PrEP among BLMSM, our study revealed an imbalance where barriers were more prominent in retrospective accounts than the specific assets and resources participants drew upon to overcome them. Future research should prioritize and more explicitly elicit multilevel resilience processes across the PrEP care continuum. While our findings depicted how BLMSM successfully sustained PrEP engagement despite multilevel challenges, targeted exploration of under-emphasized resilience mechanisms could identify scalable intervention targets to support PrEP persistence among BLMSM.

Additionally, future research should leverage patient journey mapping to anticipate and evaluate next-generation PrEP options, including twice-yearly LAI and forthcoming modalities. Journey maps can be used prospectively to examine how new products intersect with existing routines, structural constraints, and clinic workflows, and to identify points where they reduce or unintentionally introduce friction across awareness, initiation, maintenance, and re-engagement. Studies should examine how patient-level resilience processes (e.g., coping strategies, motivation, problem-solving) interact with clinic- and system-level programmatic responses when BLSMM encounter common disruptions, such as gaps in insurance coverage, pharmacy denials, clinic scheduling constraints, and medical burnout. Implementation science-focused research is also needed to evaluate which specific combinations of practice changes, staffing models, and financing approaches are most effective for supporting sustained PrEP use and rapid re-initiation after unintentional lapses (68, 69). These efforts should be reproduced across diverse clinical settings and integrated service models with other populations disproportionately affected by the HIV epidemic, including cisgender women, transgender and gender-expansive populations, and individuals with injection drug use histories, particularly from racial and ethnic-minoritized backgrounds (70, 71).

To complement qualitative insights, future work should apply large-scale, longitudinal, and multilevel study designs to classify and quantify common PrEP engagement trajectories (e.g., via latent class methods). Surveillance efforts should employ resilience-informed (e.g., stress-coping, compensatory resilience, and risk-protective models) model-building techniques to more comprehensively identify protective mediating and moderating factors that interrupt social barriers’ stalling or falling-off effects on BLMSM's advancement across the PrEP care continuum (72–74). Lastly, triangulating surveillance studies with patient journey maps, electronic health records, pharmacy claims, and other administrative data would support the development of empirically grounded models of PrEP engagement that can inform the design and testing of interventions tailored to specific populations and contexts (75).

Beyond mapping behavioral touchpoints, these findings invite refinement of how decision-making models are used to guide PrEP interventions. Classic, stage-based and reasoned-action frameworks emphasize linear progression and largely rational, deliberative choice, whereas observed trajectories reflect cyclical movement, structural shocks, and heuristic coping that align more closely with dual-process and behavioral decision-making accounts (76). Future PrEP models should therefore integrate resilience-informed and behavioral decision-science perspectives to capture how people reweigh risks and benefits over time, drawing on self-perceived representations of HIV vulnerability, and adapting choices under uncertainty, thereby better informing intervention points that support re-engagement rather than presuming stable, forward movement along the continuum.

## Conclusion

Overall, our findings converge with and deepen prior work that has emphasized potential nonlinear PrEP engagement (28), while adding granular detail on how these patterns unfold within an FQHC and how persistence is sustained despite disruptions. Building on this literature, we offer several distinct contributions, including participants' descriptions of PrEP-related fatigue and the value of more collaborative, shared decision-making approaches to patient-provider interactions that actively center BLMSM's preferences and goals. By applying qualitative journey mapping with PrEP persistent BLMSM and PrEP staff, this study moves beyond numeric representations of the PrEP care continuum to illuminate what is happening for BLMSM at each stage. We depicted how clinic-level resources helped catalyze PrEP-related resilience processes, supporting BLMSM to overcome multilevel social challenges when navigating multiple pathways across socio-cognitive, behavioral, and re-engagement stages over time. The analysis highlights patient-centered communication, financial navigation support, and dedicated PrEP coordinators as key interventions and mechanisms through which a safety net clinic can transform structural challenges, including perceived cost and insurance barriers, into opportunities for retention and re-initiation. Our study's contributions advance a resilience-informed model of PrEP engagement that can guide future research, implementation efforts, and equity-oriented program design for BLMSM and other populations disproportionately affected by the HIV epidemic.

## Data Availability

The data supporting the findings of this study consist of qualitative interview materials that may contain potentially identifiable information. Due to the sensitive nature of the data and to protect participant confidentiality, these data are not publicly available. De-identified excerpts may be made available from the corresponding author upon reasonable request and with appropriate ethical approvals.
